# Free-breathing gradient recalled echo-based CMR in a swine heart failure model

**DOI:** 10.1038/s41598-022-07611-8

**Published:** 2022-03-08

**Authors:** Craig C. Morris, Jacob Ref, Satya Acharya, Kevin J. Johnson, Scott Squire, Tuschar Acharya, Tyler Dennis, Sherry Daugherty, Alice McArthur, Ikeotunye Royal Chinyere, Jen Watson Koevary, Joshua M. Hare, Jordan J. Lancaster, Steven Goldman, Ryan Avery

**Affiliations:** 1 Department of Medicine, Oregon Health and Sciences University, Portland, OR USA; 2 MD Program, College of Medicine, University of Arizona, Tucson, AZ USA; 3 Department of Chemistry and Biochemistry, University of Arizona, Tucson, AZ USA; 4 Magnetic Resonance Research Facility, University of Arizona, Tucson, AZ USA; 5 Sarver Heart Center, University of Arizona, Tucson, AZ USA; 6 MD-PhD Program, College of Medicine, University of Arizona, Tucson, AZ USA; 7 Department of Biomedical Engineering, University of Arizona, Tucson, AZ USA; 8 Department of Medicine, University of Miami Miller School of Medicine, Miami, FL USA; 9 Department of Radiology, Northwestern University, 676 N Saint Clair, Suite 800, Chicago, IL 60611 USA

**Keywords:** Heart failure, Myocardial infarction, Cardiac device therapy, Magnetic resonance imaging, Experimental models of disease, Preclinical research, Translational research, Magnetic resonance imaging

## Abstract

In swine models, there are well-established protocols for creating a closed-chest myocardial infarction (MI) as well as protocols for characterization of cardiac function with cardiac magnetic resonance (CMR). This methods manuscript outlines a novel technique in CMR data acquisition utilizing smart-signal gradient recalled echo (GRE)-based array sequences in a free-breathing swine heart failure model allowing for both high spatial and temporal resolution imaging. Nine male Yucatan mini swine weighing 48.7 ± 1.6 kg at 58.2 ± 3.1 weeks old underwent the outlined imaging protocol before and 1-month after undergoing closed chest left anterior descending coronary artery (LAD) occlusion/reperfusion. The left ventricular ejection fraction (LVEF) at baseline was 59.3 ± 2.4% and decreased to 48.1 ± 3.7% 1-month post MI (P = 0.029). The average end-diastolic volume (EDV) at baseline was 55.2 ± 1.7 ml and increased to 74.2 ± 4.2 ml at 1-month post MI (P = 0.001). The resulting images from this novel technique and post-imaging analysis are presented and discussed. In a Yucatan swine model of heart failure via closed chest left anterior descending coronary artery (LAD) occlusion/reperfusion, we found that CMR with GRE-based array sequences produced clinical-grade images with high spatial and temporal resolution in the free-breathing setting.

## Introduction

Nearly half the population in the United States is projected to have some form of cardiovascular disease by 2035 with the annual total healthcare cost expected to reach over $1 trillion[^1^]. CHF is the leading cause of deaths attributable to cardiovascular disease and it remains the leading cause of death in the modernized world^2^. CHF is a clinical syndrome often characterized clinically by dyspnea, fatigue, and progressive pulmonary and lower extremity edema^3^. Although currently approved therapies have provided a clear benefit to those suffering from CHF, the 5-year mortality rate remains approximately 40–50%^4,5^. Thus, an opportunity is present for additional basic research to discover novel pharmacologic and biologic therapies, with the aim of improving the outcomes in patients with CHF.

The translation of experimental therapies into effective and approved treatment options requires validation in large animal models that mimic human cardiac pathophysiology^6,7^. Small animal models, such as murine and rat models, provide important insights into the mechanisms of CHF. However, significant physiologic differences exist between small animals and humans including heart rate, oxygen consumption, adrenergic receptor ratios, myocardial contractile protein expression, cardiomyocyte ion channel phenotype, cellular response to heart failure, and phenotypic differences between stem cells limiting the interpretation and translation of results from small animal models to humans^6,8–11^. Large animal models, and particularly porcine models, more closely approximate human physiology, anatomy, and immunology which makes them useful in the further development of preclinical therapies for heart failure^12–14^. Furthermore, porcine coronary vasculature is similar to that of humans^12,13^. The left anterior descending artery (LAD) provides approximately half of the blood supplied to the left ventricle (LV) and its occlusion results in ischemia of the anteroapical, lateral, and septal walls of the LV which correlate in size and distribution to humans that have LAD occlusion^15,16^. Furthermore, post-infarct LV myocardial remodeling of the heart in swine models closely resembles what occurs in humans^17,18^.

Imaging plays a critical role in the characterization of baseline and post-infarct cardiac function making it necessary for the evaluation of potential heart failure therapies. CMR is particularly useful for heart failure models as it allows for noninvasive assessments of multiple reference standard cardiac parameters to assess systolic and diastolic function including, ventricle volumes, ejection fraction, and filling rates^19,20^. Furthermore, CMR provides quantitative assessments of both myocardial mass and infarction mass as well as estimation of geometric remodeling and scar reduction^21,22^.

In swine models, there are well-established protocols for creating closed-chest MI utilizing cardiac catheterization with coronary angiography. Such a protocol published in 2012 outlined each component of the process, providing a comprehensive outline for fluoroscopy, surgery, electrocardiography, and CMR^15^. However, since its publication, advancements have been made in CMR technology and techniques, allowing for research-grade imaging to be comparable or better than clinical-grade. The previously described CMR methods utilize breath-holding or ventilator gating techniques which have been found to distort anatomy because of breath to breath variation, and cause problems with vital sign stability and end tidal CO_2_ increasing the risk for arrhythmia^23–26^.

This protocol outlines the novel use of smart-signal averaging GRE-based array of pulse sequences in nine Yucatan mini swine before and 1 month after 90 min of ischemia reperfusion of the LAD providing both high spatial and temporal resolution imaging in the free-breathing setting. Finally, we outline post-imaging analysis utilizing CMR acquired data including our use of the American Heart Association 17-segment model of infarct sizing standardization^27^ and calculation of diastolic and systolic left ventricular wall stress^28^. This new technique will be widely valuable in cardiovascular disease research in animal models as it outlines state-of-the-art CMR methods allowing for improved evaluation of cardiac function and anatomy. This methodology will be particularly useful for the evaluation of pre-clinical therapies for cardiovascular disease.

## Methods

Research oversight and approval was conducted by University of Arizona’s Institutional Animal Care and Use Committee. University Animal Care provided veterinary care, husbandry and surgical oversight within their facilities that are accredited and registered by the Association for Assessment and Accreditation of Laboratory Animal Care International (AAALACi Number (accredited since 1969): 000163, Continuing Full Accreditation (effective March 4, 2020)), Public Health Service (NIH/OLAW)(PHS Animal Welfare Assurance Number: D16-00159 (A-3248-01), Effective August 8, 2019, Expires August 31, 2023) and the United States Department of Agriculture (USDA Animal Research Facility Registration Number: 86-R-0003, Expires August 24, 2022). All experiments and methods were performed in accordance with the National Institute of Health’s “Guide for the Care and Use of Laboratory Animals” and in compliance with Institutional Animal Care and Use Committee-approved -protocols at the University of Arizona Animal Care Program (number 17-259, approved 06/14/2017). The methods and results of this study are reported in accordance with ARRIVE guidelines.

Nine male Yucatan mini swine weighing 48.7 ± 1.6 kg at 58.2 ± 3.1 weeks old underwent 90 min of occlusion/reperfusion of the left anterior descending coronary artery and were evaluated with the below detailed CMR methods at baseline and 1-month post MI.

### Animal preparation

The animals were *nil per os* (NPO) 12 h prior to surgery with water ad libitum and were delivered awake to the MRI preparation suite. In brief, the animals were sedated with ketamine 11–33 mg/kg and/or Midazolam 0.1–0.5 mg/kg delivered intramuscularly and sedated with 5% Isoflurane and 2–4 l/min of Oxygen via a nose cone. After the anesthetic plane is reached, the animal is intubated, and isoflurane was reduced to a maintenance level. Ophthalmic ointment was applied to prevent eye dehydration and an intravenous catheter was placed in an ear vein to deliver isotonic fluids at 5–10 ml/kg/h. the respiration rate, patter, mucus membrane condition, pulse oximetry, heart rate, and temperature were monitored during preparation. A ventilator was not used to prevent extreme diaphragm movement and image quality disruption.

Once anesthetized, the animals were transported to the CMR suite and all metal and electronic monitoring devices were removed. The swine was positioned in the dorsal recumbent position in a Siemens Skrya Magnetom 3 Tesla MRI system (Skrya Magnetom, Erlangen, Germany) with towel rolls to maintain stability while the head was kept accessible for extended ventilation tubes supplied by an MRI compatible ventilator. Intravenous contrast and saline were connected to Bracco Empower MR injector (Milan, Italy) for dynamic perfusion required later in the study. Physiological monitoring, End Tidal CO2, SPO2, EKG, respirations and blood pressures were monitored by veterinary staff and used by the MRI scanner for gating purposes by a Philips Expression IP5 (Koninklijke Philips, NV USA). Finally, a standard 18 channel phased array flexible body coil is conformed around the chest, being centered on the heart region, and the animal is carefully fed to isocenter with care given to not dislodge any of the apparatus.

After the imaging study, the animals were removed from the MRI unit, returned to the preparation suite, recovered to consciousness, and extubated. Observation continues until the animal can safely stand unsupported in the transport cart for transport back to University Animal Care where they were monitored visually for stability and activity (Supplementary Information).

### CMR image sequences acquisition

Nine standard two-dimensional turbo flash (TFL2D) scout images were acquired in 3 orthogonal planes to begin localization with a large 400 mm × 400 mm field of view (FOV), a thick 8 mm slice with 50% gap, resulting in 2.1 mm × 2.1 mm × 8 mm voxels. A second series, to acquire a 2 chamber view of refined orientation scout images were placed in the LV parallel to the septal wall with a smaller rectangular FOV of 274 mm × 300 mm and contiguous 8 mm slices for positioning 2 pseudo–short Axis (SA) sequences. One TFL2D with 15, 5 mm contiguous slices was acquired for further refining of the subsequent functional 2 chamber, 3 chamber and 4 chamber views. Additionally, a 2.3 s True Fast Imaging was obtained with steady-state free precession (SSFP) sequence with Cardiac B0 shim box adjusted tightly to the heart which was an inherited property of the later fast perfusion sequences. While the time to acquire this image set was not optimized for first pass myocardial perfusion images, it was duplicated for later use.

The SAx stack, 2 chamber, 3 chamber, 4 chamber, and left ventricular outflow track (LVOT) functional scans were collected with 25 calculated phases of standard retroactively reconstructed cine TFL2D sequences with radio frequency (RF) spoiling and Phase Encode (PE) rewinding in segments of 4–7 based on heart rate acquired at each slice location with 0.8 mm × 0.8 mm × 4 mm voxels using 45% phase oversampling, a repetition time (TR) of 23.53 ms, an echo time (TE) of 2.88 ms, flip angle (FA) of 15° and 3 averages to mitigate respiratory motion were uniquely acquired during free breathing.

Dark Blood Soft Tissue Inversion Recovery Turbo Spin Echo (STIR DB) sequences were performed in a similar orientation to the functional sets and were utilized to evaluate increased water content in myocardial tissue which is indicative of inflammation and is often not obvious in the cine image sets. TR is dependent on heart rate, and the target recovery time is approximately 3 s, with TE of 48 ms. These sequences are achieved with cardiac gating using 2–4 trigger pulses, depending on average heart rate. A slice selective inversion pulse of 160 m was used to achieve fat suppression and 10 long-term averages were used to mitigate respiratory motion. Scan time is just over 3 min based on heart rate with integrated parallel imaging technique (iPAT) generalized auto-calibrating partially parallel acquisitions (GRAPPA) set to 2 times (a factor of 2).

**Figure 1:**
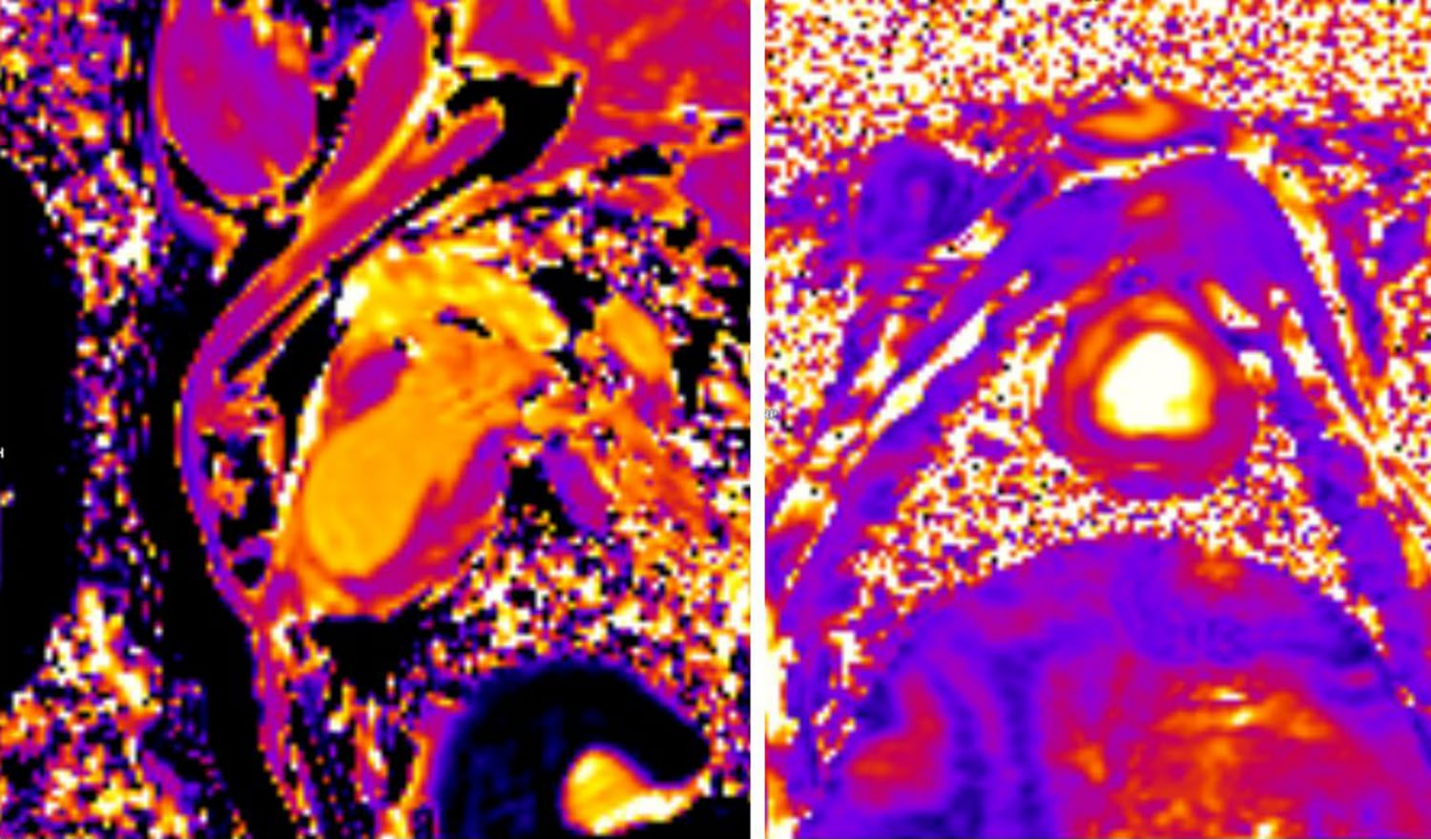
T1 and T2 Mapping 1-month post-MI acquired using Myo-Maps sequences (siemens Healthineers) in SAx and 2 chamber views. The sequence is a TFL2D variant BEAT map with rectangular FOV of 2.49 mm × 1.88 mm × 8 mm voxels.

Quantitative T1 and T2 mapping was also acquired using the proprietary Myo-Maps sequences (Siemens Healthineers) in SAx and 2 chamber views, shown in Fig. 1. The sequence is a TFL2D variant called BEAT map using rectangular FOV of 2.49 mm × 1.88 mm × 8 mm voxels. 8 mm grid tagged fast low angle shot (FLASH) sequences were also programmed with the previously positioned 2–4 chamber views to assess wall movement. These were done with a 250 mm FOV with 80% phase resolution resulting in an acquisition matrix of 115 × 176. TR = 32 ms, TE = 2.6 ms, FA = 10 degrees, 6 mm slice and 4 averages for a 2–3 min scan, again based on heart rate.

After a single measurement test scan of the dynamic TFL perfusion sequence verifies positioning with no phase wrap in any of the orientations, 0.2 mmol/kg of MultiHance gadobenate contrast (Bracco) at 2.0 ml/s was injected as 10 of 150 measurements were completed for visualization of the contrast uptake and myocardial filling. The perfusion sequences were comprised of 3 slices positioned at the best 2, 3 and 4 chamber cine locations. Data collection was adjusted so that all three gated slices fit within one RR. The voxels were interpolated to 1.6 mm × 1.6 mm with a 9 mm slice thickness using a TR controlled by heart rate but approximately 123 ms, TE of 1.02 ms achieved with a 2.1 ms echo spacing by a bandwidth set at 1008 Hz/Pixel.

While waiting for Delay Enhanced contrast uptake standard flow quantitative BEAT_FQ FLASH were acquired at the mitral valve, pulmonary artery, and aortic root using one 4 mm slice positioned to measure through-plane flow. These were retroactively reconstructed with 144 mm phase FOV 210 read FOV and 118 × 192 acquisition matrix TR approx. 21 ms and TE of 3.02 ms with PAT 2 at approximately 2 min each depending on HR.

With swine’s metabolism of contrast being faster than humans, an Inversion recovery Time scout (TI) was collected at roughly 6 min post injection to plan the delay needed to null the myocardium. This sequence runs 20 measurements and sweeps the TI from 100 to 645 ms. The TI was chosen anticipating an increase needed every several minutes. Delay enhancement scans are collected with the previously defined orientations, SAx stack, and 2–4 chamber views. These were 1.35 mm × 1.35 mm × 4 mm voxels collected with a gated TFL with a non-selective inversion pulse and 10-degree FA.

### Placement of internal cardiac monitors (ICM)

The ICM was placed 1 month prior to the MI procedure. After completing the animal preparation process outlined above, the left scapular area was clipped and scrubbed for aseptic surgery. A 1″ incision was made laterally along the left scapula and gentle dissection is used to widen the subcutaneous tissue enough to accommodate the 44.8 mm sterile ICM device. A 2-0 absorbable suture was used to close subcutaneous layers and a 3-0 absorbable suture was used for subcuticular closure with surgical glue. Bupivacaine (2 mg/kg) and/or lidocaine (4–6 mg/kg) local and regional nerve block (inverted L block) were used at incision sites after closure. ICM data is quickly collected using a reader placed over the device.

### LAD occlusion/reperfusion

After completing the animal preparation process outlined above animals were connected to a monitor for real-time respiration rate, pulse oximetry, heart rate and temperature monitoring during the procedure. Groin areas were clipped and scrubbed for aseptic surgery and the animals were placed in dorsal recumbency with front legs folded and tied caudally along ribs, with rear legs retracted and tied caudally.

The percutaneous groin access site was prepared by nicking the skin with a 10 or 15 blade scalpel. A 17–19 g percutaneous needle was then inserted into the femoral artery or femoral vein until blood returned was observed. A 0.35 J-wire is inserted through the needle, advanced into the vessel and the needle removed. An 8-11F introducer dilator and sheath were advanced over the wire. Blood reflux was confirmed, the dilator and guide wire were removed, and the introducer was flushed with heparinized saline then secured with 2-0 suture. A bolus of heparin (100–200 USP/kg) was used to prevent thromboembolism.

Lidocaine (2–4 mg/kg) was administered and repeated every 20–60 min as needed to prevent arrythmia during the procedure. Additionally, an IV pump 20–60 mcg/kg/min of lidocaine was started. If needed, amiodarone was administered by bolus, up to 2 times using 150–300 mg first dose and 75–150 mg second dose with infusion using a standard IV pump at 1 mg/min.

**Figure 2:**
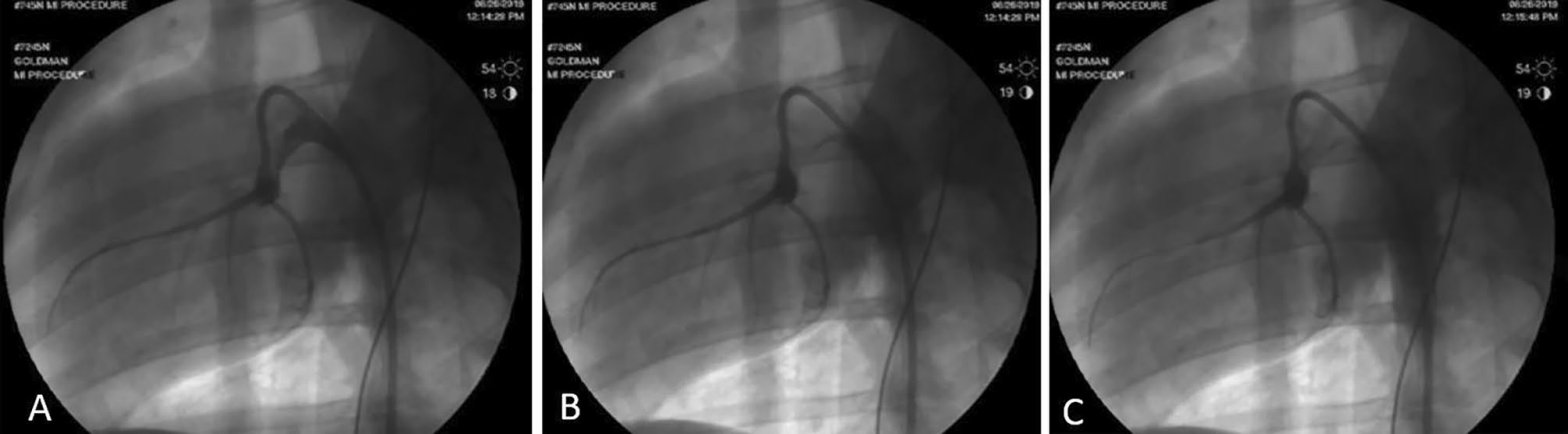
(**A**–**C**) Cardiac angiographic images in left anterior oblique (LAO) views showing placement of catheters and guide wires for induction of MI. (**A**) Guide wire with contrast agent visualizing LAD and circumflex arteries. (**B**) Deflated balloon catheter over guide wire. (**C**) Inflated balloon catheter over guide wire occluding the LAD.

An AL 0.75 or AL 1.0 guide catheter was advanced to the left coronary artery. An angiogram with contrast was performed (Fig. 2A), using an Oxilan 300 contrast agent through the guide catheter. A 0.14 guide wire was advanced through the guide catheter to the distal LAD coronary artery and advanced nearly to the apex (as shown by Fig. 2B). A percutaneous transluminal coronary angioplasty (PCTA) balloon (2.0–3.75 mm × 12-15 mm) was advanced on the guide wire until just past the first or second diagonal branch, isolating the lower 1/3 of the left ventricle (Fig. 2C). The balloon was inflated to the appropriate pressure using an inflation device and a timer was started, while contrast was flushed to confirm complete occlusion. Pressure of the balloon was adjusted to ensure complete occlusion.

The LAD was occluded for 90 min. Contrast boluses were performed again at 45 min and 90 min to confirm continuous occlusion. The balloon was then deflated, and another contrast bolus performed to confirm reperfusion of the LAD. All catheters, guide wires and sheaths were then removed.

Pressure was applied to the percutaneous access sites as the sheaths were removed, holding heavy pressure for 10 min, medium pressure for 5 min and light pressure for 5 min to help prevent hematoma formation. The tissue layers and the skin were closed with absorbable suture and surgical glue followed by local Bupivacaine (2 mg/kg) and/or Lidocaine (4–6 mg/kg) injection at incision sites after closure.

### Ethics approval and consent to participate

Research oversight and approval was conducted by University of Arizona’s Institutional Animal Care and Use Committee. University Animal Care provided veterinary care, husbandry and surgical oversight within their facilities that are accredited and registered by the Association for Assessment and Accreditation of Laboratory Animal Care International (AAALACi Number (accredited since 1969): 000163, Continuing Full Accreditation (effective March 4, 2020)), Public Health Service (NIH/OLAW)(PHS Animal Welfare Assurance Number: D16-00159 (A-3248-01), Effective August 8, 2019, Expires August 31, 2023) and the United States Department of Agriculture (USDA Animal Research Facility Registration Number: 86-R-0003, Expires August 24, 2022).

### Arrive reporting statement

The methods and results of this study are reported in accordance with ARRIVE guidelines.

### Consent for publication

All authors give consent for publication.

## Results

Nine male Yucatan mini swine weighing 48.7 ± 1.6 kg at 58.2 ± 3.1 weeks old underwent the procedure described. Table 1 summarizes the data obtained from CMR with all results reported as mean ± SEM. The paired percent change was calculated for each animal then averaged. Additionally, a paired t-test statistic was calculated to determine the P value. The average LVEF at baseline was 59.3 ± 2.4% and decreased to 48.1 ± 3.7% 1-month post MI, resulting in an average of − 18.0 ± 6.7 mean percent decrease in LVEF (P = 0.029). Average EDV at baseline was 55.2 ± 1.7 ml and increased to 74.2 ± 4.2 ml at 1-month post MI resulting in an average increase of 16.8 ± 6.7% (P = 0.001). The average end-systolic volume (ESV) at baseline was 22.6 ± 1.8% and increased to 39.1 ± 4.2% at 1-month post MI resulting in an average increase of 78.4 ± 22.0% (P = 0.005). Additionally, LV myocardial mass was determined. The left ventricular mass at baseline was 64.5 ± 1.8 g and increased to 76.1 ± 3.5 g at 1-month post MI. The CMR estimated LV percent scar at 1-month post MI was 23.2 ± 3.7%.

**Table 1:** LV CMR parameters before and 1-month post MI.

	Baseline	1-month post MI	Avg percent change (%)	Paired t-test
Swine mass (kg)	48.7 ± 1.6	48.8 ± 1.4	0.6 ± 1.9	n/a
Heart rate (bpm)	98.7 ± 6.8	91.7 ± 8.5	− 6.6 ± 6.7	0.321
LVSP (mmHg)	85.2 ± 4.1	77.9 ± 3.7	− 8.0 ± 4.0	0.090
LVDP (mmHg)	11.6 ± 1.4	8.8 ± 2.1	− 5.5 ± 27.4	0.427
EDV (mL)	55.2 ± 1.7	74.2 ± 4.2	33.8 ± 5.1	0.001
ESV (mL)	22.6 ± 1.8	39.1 ± 4.2	78.4 ± 22.0	0.005
SV (mL)	32.7 ± 1.4	35.1 ± 2.7	8.0 ± 7.8	0.358
EF (%)	59.4 ± 2.4	48.1 ± 3.7	− 18.0 ± 6.7	0.029
CO (L/min)	3.2 ± 0.2	3.2 ± 0.3	2.5 ± 9.9	0.973
LV mass (g)	64.5 ± 1.8	76.1 ± 3.5	17.6 ± 17.6	0.091
LV percent scar (%)	0	23.2 ± 3.7	n/a	n/a
DWS ((dyne/cm^2^) × 1000)	7.6 ± 1.0	15.5 ± 3.6	148 ± 69.7	0.056
SWS ((dyne/cm^2^) × 1000)	31.6 ± 3.5	36.2 ± 4.0	17.1 ± 11.6	0.221
RAP (mmHg) n = 6	6.3 ± 0.8	5.7 ± 0.9	− 2.8 ± 23.3	0.603
PASP (mmHg) n = 6	23.7 ± 3.1	18.2 ± 1.4	− 5.4 ± 35.2	0.205
PADP (mmHg) n = 6	11.4 ± 2.4	7.4 ± 1.8	− 65 ± 113.2	0.373
PCWP (mmHg) n = 6	8.6 ± 2.2	4.5 ± 1.5	− 24.9 ± 74.4	0.205

Results are shown as average ± SEM (n = 9, unless otherwise noted in table). Paired percent changes were calculated then averaged.

*EDV* end diastolic volume, *ESV* end systolic volume, *LVSP* left ventricular systolic pressure, *LVDP* left ventricular systolic pressure, *SV* stroke volume, *EF* ejection fraction, *CO* cardiac output, *LV* left ventricle, *DWS* diastolic wall stress, *SWS* systolic wall stress, *RAP* right atrial pressure, *PASP* pulmonary artery systolic pressure, *PADP* pulmonary artery diastolic pressure, *PCWP* pulmonary capillary wedge pressure.

**Figure 3:**
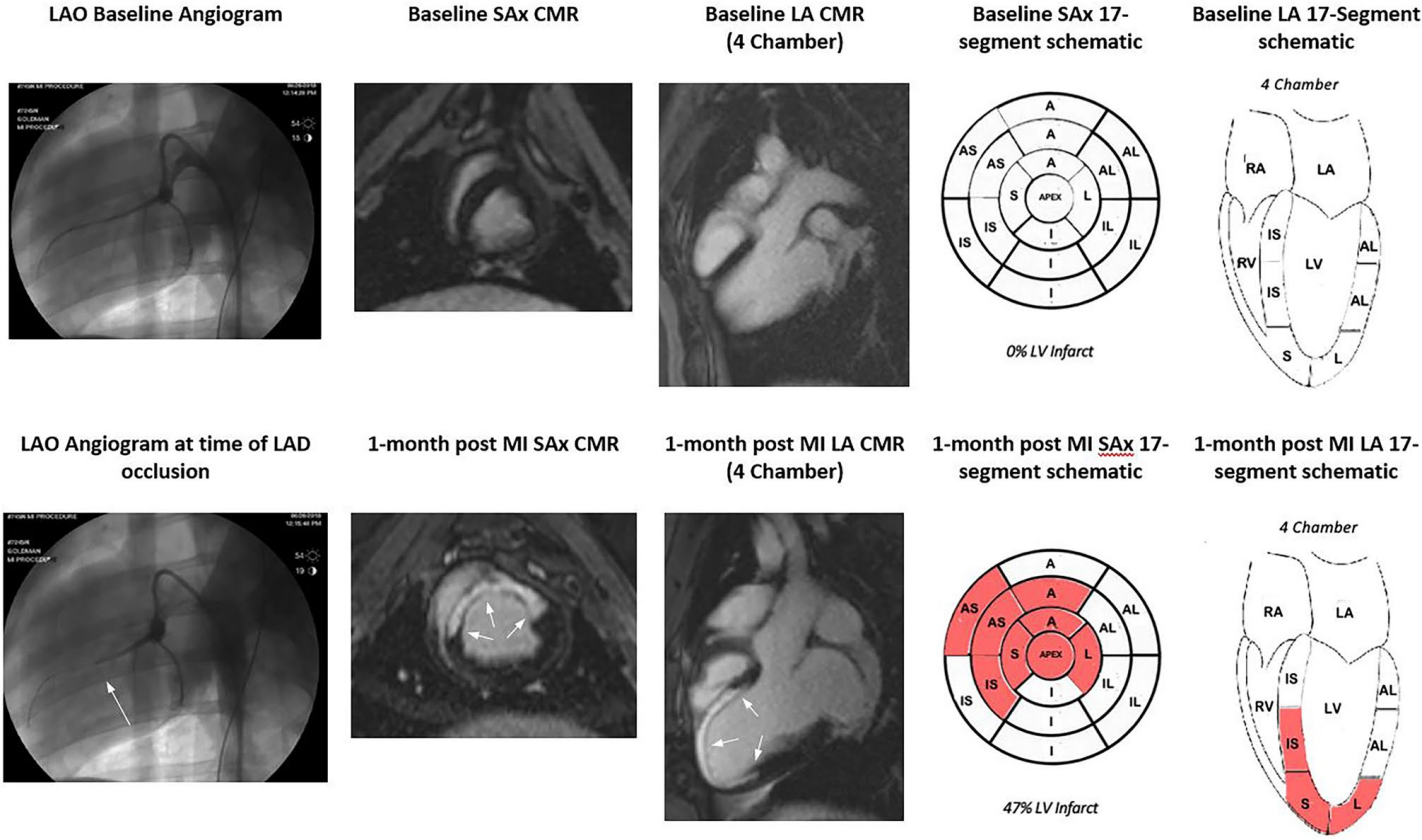
The images on the top row show pre-infarct (baseline) angiography, SAx CMR, LA 4 chamber CMR, SAx 17-segement schematic, and LA 4 chamber 17-segment schematic (from left to right). The images on the bottom row show angiography after occlusion of the LAD just distal to the first diagonal branch and 1-month post MI images of SAx CMR, LA 4 chamber CMR, SAx 17-segement schematic, and LA 4 chamber 17-segment schematic (from left to right). In the 17-segment schematics, the letters denote different anatomical areas of the heart with *AS* anteroseptal, *IL* inferolateral, *A* anterior, *I* inferior, *IS* inferoseptal, *AL* anterolateral. The 17-segment SAx wheel shows the apex at center most portion of the wheel and the base segments at the outermost portion of the wheel. Quantification of myocardial ischemic injury is shown in red. Average infarct percent volume (%) is denoted below the 17-segment schematic.

**Figure 4:**
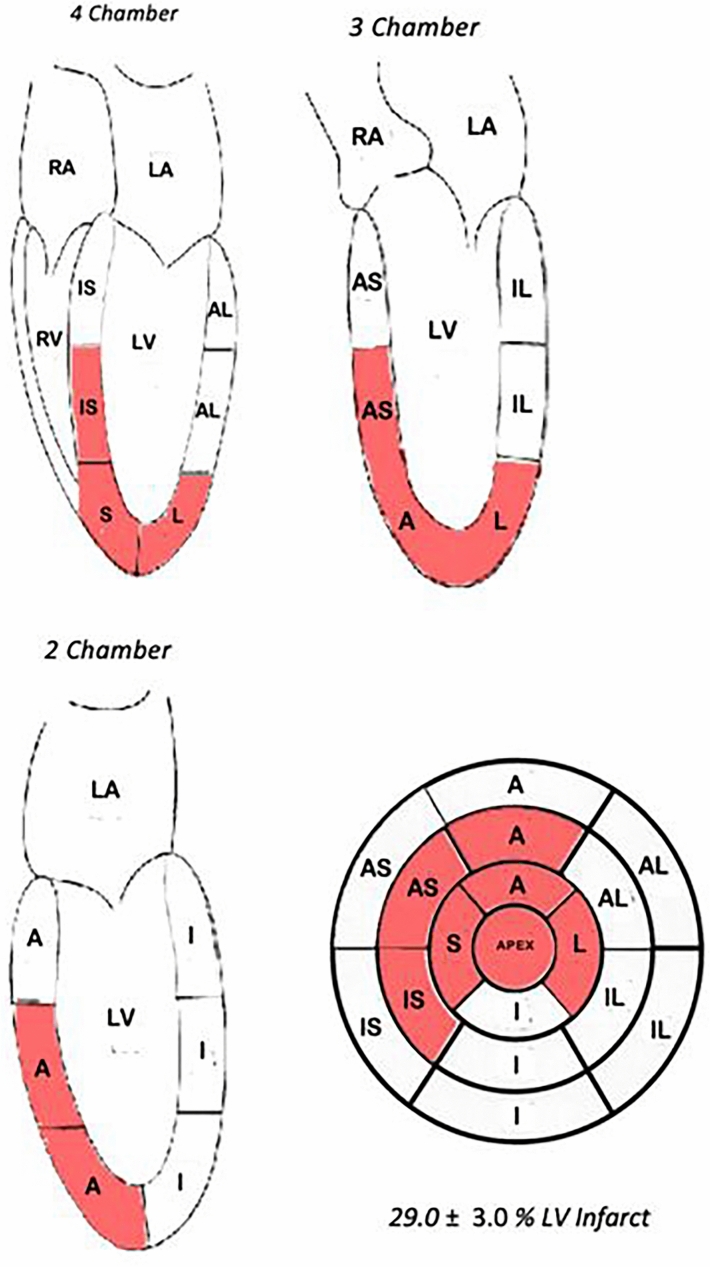
In the 17-segment schematics, the letters denote different anatomical areas of the heart with *AS* anteroseptal, *IL* inferolateral, *A* anterior, *I* inferior, *IS* inferoseptal, *AL* anterolateral. The 17-segment SAx wheel shows the apex at center most portion of the wheel and the base segments at the outermost portion of the wheel. Segments with myocardial ischemic injury are shown in red. The average estimated infarct percent volume (%) is denoted below the 17-segment wheel.

Figure 3 shows an overview of the process including angiogram at baseline and occlusion, CMR images in LA and SAx orientation at baseline and 1-month post MI, and 17 segment infarct sizing. The myocardial ischemic injury locations at 1-month post MI are depicted in both long axis (LA) and short axis (SA) orientations in Fig. 4. Utilizing 17-segment infarct sizing, the average LV percent scar was 29. ± 3.0%.

Diastolic wall stress at baseline was 7.6 ± 1.0 (dyne/cm^2^) × 1000 and increased to 15.5 ± 3.6 (dyne/cm^2^) × 1000 at 1-month post MI while systolic wall stress at baseline was 31.6 ± 3.5 (dyne/cm^2^) × 1000 and increased to 36.2 ± 4.0 (dyne/cm^2^) × 1000 at 1-month post MI (Table 1).

The CMR imaging technique described allows for highly reproducible images to evaluate cardiac function. Figure 3 shows short and long axis delayed enhancement images pre- and post-infarct and demonstrate wall thinning, chamber dilation, and scar formation of portions of the LV at 1-month post-MI.

## Discussion

This protocol provides a consistent and reliable method to obtain serial cardiac magnetic resonance imaging in Yucatan mini swine before and after 90 min of balloon occlusion/reperfusion of the LAD. While previously described CMR techniques allow for characterization of cardiac structure and function, this represents an updated methodology producing clinical-grade images and enhanced myocardial characterization for more accurate evaluation of heart failure models and pre-clinical therapies.

CMR with smart-signal averaging GRE-based array of pulse sequences allowed for both high spatial and temporal resolution imaging in the free-breathing setting. The use of GRE-based imaging allows for a reduction in various artifacts, including dephasing artifact related to turbulent flows in the hyperdynamic swine hearts, susceptibility artifact related to implanted metal artifact such as monitoring probes or sternal wires, and most importantly, the avoidance of off-resonance artifacts which often require repeat frequency scouts and shimming during the exam which can significantly increase scan time. With the multi-averaged technique, the long axis stack of 3 cine SSFP images take 4–6 min while the short axis (SA) stack evaluating the bot ventricles took between 8 and 12 min. Breath holding has been noted to result in additional issues with variations in cardiac volume and contraction resulting in decreased in resolution as well as vital sign stability, end tidal CO2 variation, and heart rate variability^23–26^.

While the protocol provides a robust evaluation of myocardial size, function, and tissue characterization with superior temporal and spatial resolution that can be performed on anesthetized swine, it does have limitations. Particularly, while cardiac MRI is an ever-growing technique, its use is often limited to academic centers given the infrastructure needed for maintaining an MRI that can be used for both clinical and research purposes along with the computer hardware and software requirements to perform quantitative image analysis. Furthermore, swine models require facilities, staff, and procedures to comply with all federal and state guidelines concerning the use of animals in research and teaching, as defined by the Guide for the Care and Use of Laboratory Animals. Another important limitation of this study is that the results obtained utilizing this novel protocol were unable to be directly compared to conventional MRI protocols thus the degree of improvement from previous methods is unable to be objectively quantified in this manuscript.

This CMR protocol is widely applicable to cardiovascular disease research in animal models as it outlines state-of-the-art CMR methods allowing for improved evaluation of cardiac function and anatomy. It will be particularly useful for the evaluation of pre-clinical therapies for cardiovascular disease including but not limited to cell therapy, drug therapy, and device therapy. Additionally, this protocol will be useful for research aimed at better understanding cardiovascular disease pathophysiology, biophysics, and computer modeling.

The American Heart Association 17-segment model analysis provides a means of standardizing and comparing infarct measurements obtained from various modalities such as echocardiography, CMR, cardiac CT, and positron emission computed tomography^27^. Of note, the American Heart Association 17-segment model does tend to overestimate infarct sizing as there is no way to represent a partial infarct in one segment. This was demonstrated in this study as the CMR average measured LV percent scar was 23.2 ± 3.7% compared to the 17-segment computed LV infarct percent of 29 ± 3.0%.

Wall stress has been shown to be an important predictor of mortality in myocardial infarction^29^ and heart failure^30^ patients. It is provided here to demonstrate another important metric that can be obtained through analysis of CMR data acquired using GRE based array sequences in the free-breathing setting.

## Conclusion

In a Yucatan swine model of heart failure with closed chest LAD occlusion/reperfusion, we find that this novel method of CMR with GRE based array sequences produced clinical-quality images with high spatial and temporal resolution in the free-breathing setting.

## Supplementary Information

Supplementary Information.

## Data Availability

The datasets used and/or analyzed during the current study are available from the corresponding author on reasonable request.

## Abbreviations

CHF: Congestive heart failure
CMR: Cardiac magnetic resonance
CO: Cardiac output
DWS: Diastolic wall stress
LAD: Left anterior descending artery
EDV: End-diastolic volume
EF: Ejection fraction
ESV: End-systolic volume
FA: Flip angle
FLASH: Fast low angle shot
FOV: Field of view
GRAPPA: Generalized autocalibrating partially parallel acquisitions
HR: Heart rate
ICM: Internal cardiac monitor
iPAT: Integrated parallel imaging technique
IVC: Inferior vena cava
LAD: Left anterior descending coronary artery
LAX: Left anterior oblique
LAx: Long axis
LV: Left ventricle
LVEF: Left ventricular ejection fraction
LVOT: Left ventricular outflow track
LVSP: Left ventricular systolic pressure
NPO: Nil per os
PADP: Pulmonary artery diastolic pressure
PASP: Pulmonary artery systolic pressure
PCWP: Pulmonary capillary wedge pressure
PTCA: Percutaneous transluminal coronary angioplasty
PE: Phase encode
RAP: Right atrial pressure
RF: Radio frequency
SAx: Short axis
SSFP: Steady-state free precession
STIR: DB dark blood soft tissue inversion recovery turbo spin echo
SWS: Systolic wall stress
TE: Echo time
TFL2D: Two-dimensional turbo flash
TI: Inversion recovery time scout
TR: Repetition time
