# Supplementary material for: Free-breathing gradient recalled echo-based CMR in a swine heart failure model
Source: Sci Rep. 2022 Mar 8;12:3698. doi: 10.1038/s41598-022-07611-8 (PMC8904633; doi:10.1038/s41598-022-07611-8)
Supplement: Supplementary file 1 — Supplementary Information. [file 41598_2022_7611_MOESM1_ESM.docx]

**Supplemental Information:**

**Materials:**

***Reagents***

- Ketamine 11-33 mg/kg (Sedation)
- Midazolam 0.1-0.5 mg/kg (IM) (Sedation)
- Isoflurane mask 2-5% (Induction)
- Isoflurane endotracheal tube 1.0-2.5% (Anesthesia)
- MultiHance® gadobenate dimeglumine contrast agent (~0.1 mmol/kg)
- Plasmalyte or similar fluids
- Heparinized saline
  - IV flush (5-10 USP(iu)/ml)
- Heparin (100-200 USP(iu)/kg)
- Lidocaine (2-4 mg/kg)
- Oxilan® injection 300 mgl/ml
- 15%-30% hypertonic saline
- Analgesia
  - Buprenorphine SR (0.12-0.27 mg/kg; SC)
  - Carprofen or ketoprofen (2-4 mg/kg; IM, SC)
- Amiodarone
- Bupivacaine (2 mg/kg)

***Equipment***

- MRI
  - Intravenous (IV) catheter (20-22 g)
  - Siemens Skrya Manetom 3 Tesla MRI system (Erlangen Germany), shown in Figure 13.
  - MRI compatible ventilator
- Myocardial Infarction
  - Intravenous (IV) catheter (20-22 g)
  - Scalpel blade
  - 17-19 g percutaneous needle
    - - 0.35 mm J-wire, 45-60 cm (venous/arterial access)
  - 8-11F introducer dilator and sheath
  - IV pump(s)
  - 2-0 silk suture
  - Absorbable Sutures (Vicryl™ or similar)
  - Surgical Glue
  - Guide Catheters
    - IMA (90 cm)
    - AL 0.75 (90 cm)
    - AL 1.0 (90 cm)
  - Guide Wires
    - 0.35 mm J-wire
      - 120-150 cm
    - 0.14 mm wire
  - Inflation device
  - PTCA Balloon (2.0-3.75 mm X 12-15 mm)
  - Occlusion Balloon 7F (33 mm X 65 mm)
  - AD Instruments Equipment and Software
    - PowerLab 16/35 unit
    - MPVS Ultra PV Unit
    - Dual Bio Amp with 3 lead ECG cable
    - LabChart Software
    - Millar Ventri-Cath-507 (5F, 12E, 7 mm, DField, Pigtail, 122 cm)
  - Edwards Lifesciences Vigilance Monitor (Cardiac Output)
    - Swan-Ganz catheter 7F (131F7)
  - Contrast/Saline Manifold Setup
  - Gateway Advantage Y-Adapter
  - Stop-cock
- Internal Cardiac Monitor (ICM)
  - Reveal LINQ monitor
  - Absorbable Sutures (Vicryl™ or similar)
  - Surgical Glue

**Supplemental Images:**


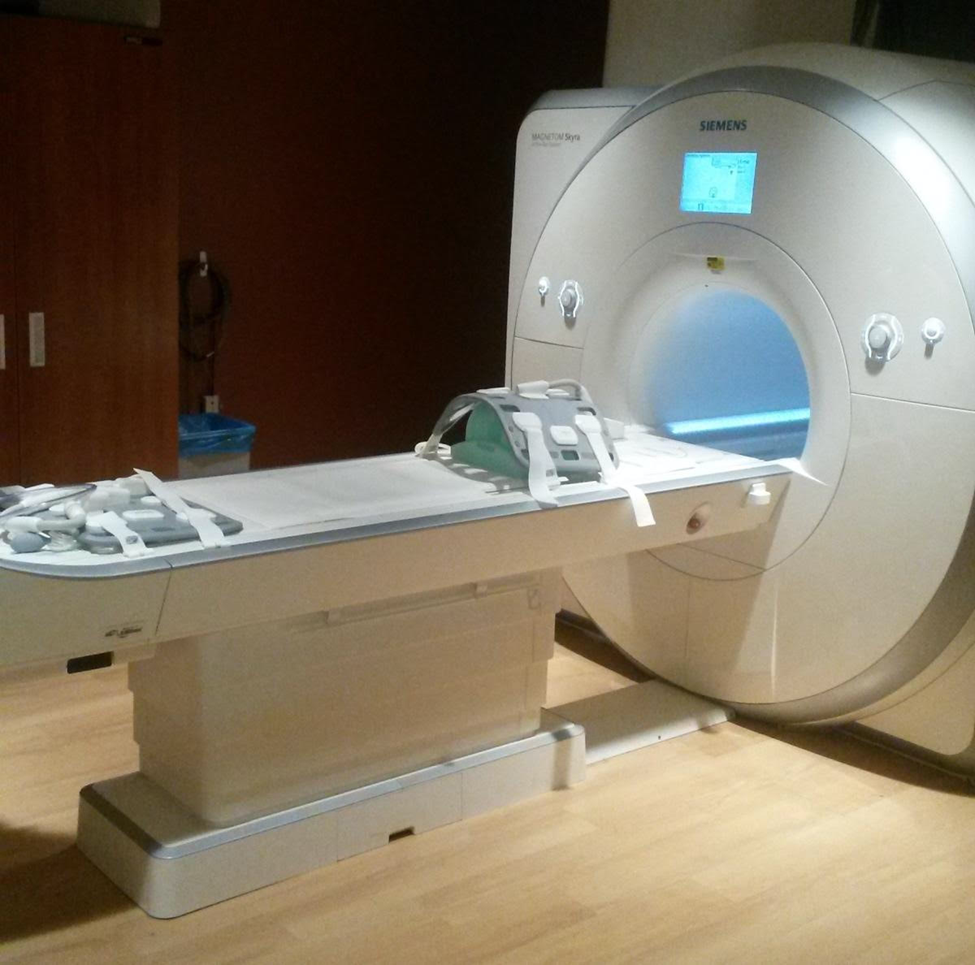


Supplemental image: Siemens Skyra Manetom 3 Tesla MRI system pictured in MRI facility**.**
